# The whole - genome expression analysis of peripheral blood mononuclear cells from aspirin sensitive asthmatics *versus* aspirin tolerant patients and healthy donors after *in vitro* aspirin challenge

**DOI:** 10.1186/s12931-015-0305-4

**Published:** 2015-12-09

**Authors:** Joanna Wieczfinska, Dorota Kacprzak, Karolina Pospiech, Milena Sokolowska, Magdalena Nowakowska, Ewa Pniewska, Andrzej Bednarek, Izabela Kuprys–Lipinska, Piotr Kuna, Rafal Pawliczak

**Affiliations:** Department of Immunopathology, Medical University of Lodz, Chair of Allergology, Immunology and Dermatology, 7/9 Zeligowskiego, 90-752 Lodz, Poland; Department of Molecular Carcinogenesis, Medical University of Lodz, Chair of Molecular Medicine and Biotechnology, Lodz, Poland; Swiss Institute of Allergy and Asthma Research (SIAF), University of Zurich, Christine Kühne-Center for Allergy Research and Education, Davos, Switzerland; Department of Internal Medicine, Asthma and Allergy, Medical University of Lodz, Lodz, Poland

**Keywords:** Aspirin – induced asthma, Aspirin, Nonsteroidal anti-inflammatory drugs, Asthma

## Abstract

**Background:**

Up to 30 % of adults with severe asthma are hypersensitive to aspirin and no unambiguous theory exists which provides a satisfactory explanation for the occurrence of aspirin-induced asthma (AIA) in some asthmatic patients. Therefore, the aim of this study was to compare the AIA expression profile against aspirin tolerant asthma (ATA) and healthy volunteers (HV) profile in peripheral blood mononuclear cells (PBMCs) after *in vitro aspirin* challenge in Caucasian population.

**Methods:**

PBMCs were separated from blood of three groups of subjects - 11 AIA, 7 ATA and 15 HV and then stimulated by either 2 μM lysine aspirin or 20 μM lysine as a control. Subsequently, RNA was isolated, transcribed into cDNA and subjected to microarray and qPCR studies. Simultaneously, protein was extracted from PBMCs and used in further immunoblotting analysis.

**Results:**

The validation of results at mRNA level has shown only three genes, whose expression was significantly altered between comprising groups. mRNA expression of *CNPY3* in PBMCs in AIA was significantly lower (-0.41 ± 2.67) than in HV (1.04 ± 2.69), (*p* = 0.02); mRNA expression of *FOSL1* in PBMCs in AIA was also significantly decreased (-0.66 ± 2.97) as opposed to HV (0.31 ± 4.83), (*p* = 0.02). While mRNA expression of *ERAS* in PBMCs was increased (1.15 ± 0.23) in AIA in comparison to HV (-1.32 ± 0.41), (*p* = 0.03). At protein level the changed expression of one protein was confirmed. Protein expression of *FOSL1* in PBMCs in AIA was both significantly lower (-0.86 ± 0.08) than in ATA (0.39 ± 0.42), (*p* = 0.046) and in HV (0.9 ± 0.27), (*p* = 0.007).

**Conclusions:**

This pilot study implies a positive association between CNPY3, ERAS, FOSL1 and aspirin-intolerant asthma, suggesting that these findings would be useful for further investigations of NSAIDs mechanism.

**Electronic supplementary material:**

The online version of this article (doi:10.1186/s12931-015-0305-4) contains supplementary material, which is available to authorized users.

## Background

Aspirin-exacerbated respiratory disease (AERD) is a distinct asthma phenotype mainly characterized by chronic eosinophilic inflammation of the upper and lower airways with symptoms that are exacerbated by aspirin and other nonsteroidal anti-inflammatory drugs (NSAIDs) [1–4].

It is estimated that 0.6–2.5 % of total population [5, 6], 5–10 % of asthmatic adults [7–9], almost 30 % adults with severe asthma [10] and just about 40 % of asthmatic adults with refractory chronic hyperplastic sinusitis [11] are hypersensitive to aspirin (ASA). Emphatically, more than 15 % of asthmatic patients are quite unaware of suffering from this intolerance [12] and only provocation tests may reveal AIA. Higher incidence of AIA was also reported in women in whom symptoms start earlier and disease course is more rapid and severe [13].

Although the exact pathomechanism of AIA still remains unknown, pathognomonic reactions to COX-1 active drugs can be attenuated by inhibitors of 5-lipoxygenase (5-LOX), type 1 receptor for cysteinyl leukotriens (cysLTR1) [14] and by drugs that block mast cells (MA) activation [15, 16]. Moreover, inhaled prostaglandin E_2_ (PGE_2_) inhibits aspirin - induced bronchoconstriction and cysLT production in subjects with AERD [17]. PGE_2_ is formed from COX-dependent conversion of arachidonic acid to PGH_2_, which is metabolized to PGE_2_ by three PGE_2_ synthases (PGESs) [18]: cytosolic PGES and microsomal PGES (mPGES-1 and mPGES-2) [19, 20]. Absence of mPGES-1 impairs the up-regulation of PGE_2_ production in mice [21]. Additionally PGES^-/-^ mice develop marked eosinophil - dominated bronchovascular cellular infiltrates with lesser numbers of neutrophiles [22, 23] and lysine aspirin (Lys-ASA) challenge caused additively releases of two markers of MC activation – histamine, mMCP-1 and cysLTs [21]. The marked depletion of residual PGE_2_ by Lys-ASA in the PGES^-/-^ mice suggests that mPGES-1 sustains PGE_2_ generation in the face of COX-1 inhibition [21]. It has been also demonstrated that platelet- adherent eosinophils and neutrophils are more frequent in the peripheral blood and sinonasal tissues from patients with AERD than in samples from aspirin tolerant controls [24]. Adherence to platelets primes granulocyte integrin function [25], chemotaxis [26] and increase susceptibility to inflammation [21]. It is probably that TP receptors are essential for platelet-adherent granulocytes to generate cysLTs by facilitating cross – talk between platelets and granulocytes [21]. Though the residual local PGE_2_ derives principally from COX-1, which may explain why only COX-1 – active drugs provoke clinical reactions [27].

It is also known that the production of 15–hydroxyeicosatetranoic acid (15–HETE) in AIA patients is 3.6 fold higher than in ATA patients [28]. The substantial source of 15–HETE in this reaction seems to be 15–lipooxygenase (15–LOX) that is controlled by COX-1 [29]. Thus, inhibition of COX-1 and disregulation of PGE_2_ production by aspirin results in activation of 15-LOX and 15-HETE production [28]. Overproduction of 15-HETE in aspirin sensitive asthmatics *inter alia* contributes to the induction of mucous glycoprotein secretion by human airway [30] and contraction of bronchial smooth muscles [31]. According to these results *in – vitro* test (ASPItest) is known that measures ASA – induced 15 – HETE in peripheral blood. ASPItest does not require special expertise, equipment and seems to be highly sensitive and specific to confirm the history of aspirin sensitivity in asthmatic patients [29].

So far, in the literature there is also a lot of data concerning genetic mechanisms suggesting the involvement of various candidate genes in the pathogenesis of AIA. Unfortunately, the majority of these results is not consistent between various populations indicating environmental factors which may predestine to development of AIA. Moreover, the likelihood that AIA is acquired in adulthood implies potential epigenetic modifications of the relevant mediator systems. Hence, it has been demonstrated that PGE_*2*_ synthase gene in nasal polyps from subjects with AERD is hypermethylated in comparison to nasal polyps from aspirin – tolerant controls [32].

The aim of this study was to explore the possible difference between aspirin-induced asthma (AIA), aspirin tolerant asthma (ATA) and HV (healthy volunteers) genetic profiles in PBMCs in Caucasian population by means of *whole genome scan* after *in vitro* aspirin challenge.

## Methods

### Study subjects

Subjects of Caucasian origin were recruited from Department of Internal Medicine, Asthma and Allergy; Medical University of Lodz; Poland. The diagnosis of bronchial asthma was based on an patient’s history, physical examination and pulmonary function tests according to Global Initiative for Asthma (GINA) 2014 guidelines. Asthmatic patients were included in the study if they met the following criteria: clinical diagnosis of asthma confirmed by bronchial hyperactivity assessed by a positive bronchodilator or methacholine test, the incidence of asthmatic attacks and no other respiratory disorders. Patients were asked to refrain from short acting bronchodilators for at least six hours before challenge.

Aspirin–sensitive asthmatic subjects were included in the study if they had a positive oral provocation test with aspirin during the last 6 months, made without the context of the study. Patients with aspirin-tolerant asthma and healthy subjects were involved in the project if they had had a negative history of aspirin or other NSAIDs hypersensitivity and had been exposed to these medicaments during at least the last six months without any adverse events before the study. The clinical profiles of asthma patients and healthy control subjects are summarized in Table 1. The study protocol was approved by the Ethics Committee of the Medical University of Lodz (permission no. RNN/107/08/KE, RNN/103/11/KE) and written consent was obtained from every subject prior to the study.

**Table 1:** Characteristics of recruited aspirin - sensitive asthmatics, aspirin - tolerant asthmatics and healthy volunteers

	Aspirin - sensitive asthmatics	Aspirin - tolerant asthmatics	Healthy volunteers
Number of subjects (n)	11	7	15
Gender (n, female/male)	6/5	3/4	11/4
Age (years, median (range))	39 (21–71)	44 (30–67)	30 (21–58)
FEV1 (% predicted)	81.45 ± 19.36	88.43 ± 7.89	
PEF (% predicted)	89.14 ± 20.24	84.64 ± 20.47	
Inhaled GCS (μg/day, mean (range))^a^	1200 (0–2400)	1120 (400–2400)	
Systemic GCS (mg/day, mean (range))^b^	0	0 (0–12)	
Current smokers (%)	27.3	14.3	
Subjects with sinusitis (%)	63.6	85.7	
Subjects with rhinitis (%)	72.7	100	
Subjects with atopy (%)	45.5	85.7	

^a^inhaled GCS were calculated as budesonide equivalents, ^b^systemic GCS were calculated as prednisone equivalents

### PBMCs isolation and incubation with lysine aspirin/lysine

Peripheral venous blood was collected before aspirin challenge. PBMCs were separated using Histopaque® 1077 solution (Sigma Aldrich, Saint Louis, MO) according to the manufacturer’s protocol, washed three times in PBS. Afterwards, the PBMCs were incubated either wuth lysine aspirin (2 μM) or lysine (20 μM) for 30 min at 37 °C. Incubation conditions for the cells were selected on the basis of previous, unpublished pilot studies. PBMC counts were not statistically different between groups before and after incubation with lysine aspirin or lysine.

### cDNA synthesis and microarray hybridization

RNA was isolated (RNAeasy Mini Kit, Qiashredder (Qiagen, Hilden, Germany) and trascribed into cDNA (ImProm II Reverse Transcription Kit (Promega, Madison, Wisconsin)), which was subjected to microarray analysis.

### Microarray procedures

Microarray flip dye experiments were performed with Human OneArray® Whole Genome Microarrays v 5.1 (Phalanx Biotech, San Diego, CA) containing 30,255 oligonucleotide probes (29,187 human genome probes and 1,088 experimental control probes) was used for gene expression analysis. Each sample was hybridized against Universal Human Reference RNA (Stratagene, La Jolla, CA, USA) provided a common denominator for accurate and reproducible comparisons of gene expression data. Single-stranded cDNA samples were labelled with Cy3 and Cy5 using ULS™ Labelling Kit (Kreatech Diagnostics, Netherlands).

Synthesis of target cDNA probes and hybridization were performed according to protocol. The preparation of a slide for hybridization included pre-wash in ethanol and pre-hybridization according to manufacturer’s protocol. Hybridization was performed in a humidity chamber filled with 2× SSPE buffer at 42 °C for 16–18 h. Post-hybridization washes were performed with the following buffers: 1× SSPE/0.03 % SDS (2 min, 42 °C), 1× SSPE (2 min, RT) and 0.1× SSPE (rinsed several times, RT).

### qPCR for candidate genes

cDNA was subjected to qPCR using the kits of primers and probes designed for the selected genes and *GAPDH* as a qPCR reference (Life Technologies, Carlsbad, CA). Assay ID and contex sequences used in this study are shown in Table 2. Each sample was measured in duplicate using TaqMan analyzer 7900 (Life Technologies, Carlsbad, CA). Using the 2^-ΔΔCt^ method, data are presented as a fold change in gene expression normalized to endogenous reference gene *GAPDH* and relative to a control (lysine-treated PBMCs). The fold change of mRNA expression in each patient was calculated by comparing RQ (2^-ΔΔCt^).

**Table 2:** qPCR probes used for expression analysis of specified genes

Gene names	Context sequence*(5’ to 3’)	Catalog number
*ALOX5*	GGAGGTCCAGCAAGGGAAACA	Hs01095330_m1
*ALOX15*	TATCTTCAAGCTTATAATTCC	Hs00609608_m1
*BMP2*	CACCATGAAGAATCTTTGGAA	Hs00154192_m1
*CNPY3*	CCAAGGGCATGTCAGAGACCT	Hs00198139_m1
*CSF1*	CATGACAAGGCCTGCGTCCGA	Hs00174164_m1
*CXCL11*	ACAGTTGTTCAAGGCTTCCCC	Hs00171138_m1
*DOCK9*	TTAAGTTGCTGCGAAACCAGA	Hs00324508_m1
*DPP9*	CTACCTGGGAATGCCATATGG	Hs00373589_m1
*ERAS*	CGAGTGTTGTGTGGGTGGGAG	Hs00742161_s1
*FOSL1*	CCCAGCAGAAGTTCCACCTGG	Hs04187685_m1
*GAB3*	ACCTGGAAAGCTGATGTAGAA	Hs00369794_m1
*MARVELD1*	GGGCCTGTAAGGTTTCCATGT	Hs00230362_m1
*PARVG*	AGCCTCCAAAGGACGTCTTTG	Hs00223323_m1
*RXRG*	CAGATCCTCAGGAAAGCACTA	Hs00199455_m1
*TLR7*	ACTAAAAATGGTGTTTCCAAT	Hs00152971_m1
*TRIP6*	TGAGGACTTTCACAGGAAGTT	Hs00377979_m1

*Assays ordered from Life Technologies are provided with context sequence surrounding the assay location. Probes contain typically 13–18 bases and the minor groove binder (5–6 bases on 3’ end stabilizing the probe)

### Protein isolation and immunoblotting analysis

Total protein was isolated utilizing RIPA lysis buffer (Sigma, Saint Louis, MO) with addition of Protease Inhibitor Cocktail (Sigma, Saint Louis, MO) according to manufacturer’s protocol and analyzed by immunoblotting method to detect selected proteins (Table 3) using 10 μg total protein per sample. Detailed immunoblotting protocol is provided in Additional file 1.

**Table 3:** Primary antibodies used for immunoblotting

Protein names	Primary antibody	Dilution	Producer	Catalog number
ALOX5	Rabbit polyclonal	1:1000	Cell Signalling	3748
ALOX15	Rabbit polyclonal	1 mg/ml	Aviva Systems Biology	ARP56030_P050
BMP2	Rabbit polyclonal	1:1000	Abnova	H00000650-D01P
CNPY3	Rabbit polyclonal	0.2 μg/ml	Aviva Systems Biology	ARP34422_P050
CSF1	Rabbit polyclonal	1/100	Abcam	ab93335
CXCL11	Rabbit polyclonal	0.2 μg/ml	Abcam	ab9955
DOCK9	Rabbit polyclonal	1/5000	Abcam	ab70272
DPP9	Rabbit polyclonal	1/2500	Abcam	ab42080
ERAS	Rabbit polyclonal	0.2 μg/ml	Aviva Systems Biology	ARP55794_P050
FOSL1	Rabbit monoclonal	1:1000	Cell Signalling	5281
GAB3	Rabbit polyclonal	1/250	Abcam	ab121311
MARVELD1	Rabbit polyclonal	1 μg/ml	Abcam	ab91640
PARVG	Rabbit polyclonal	1:1000	Abnova	H00064098-D01P
RXRG	Rabbit polyclonal	1:1000	Cell Signalling	5629
TLR7	Rabbit Polyclonal	1:5000	GeneTex	GTX125910
TRIP6	Rabbit polyclonal	0.2 μg/ml	Aviva Systems Biology	ARP51617_P050

### Statistical analysis

#### Microarray studies

For microarray studies, detection of *p values* and normalization were performed for the extracted values. Statistical significance of the microarray data was calculated by the Student’s t test – standard two-sample *t-*statistics with pooled variance. Additional statistical analysis was performed using the false discovery rate (FDR) to correct for multiple comparisons in multiple hypothesis testing. FDR of a test was defined as the expected proportion of false positives among the declared significant results [33, 34] as it is a more convenient scale to work on instead of the p-value scale [35]; it is not too conservative for microarray studies and does not lead to low sensitivity [35].

For the diagnostic values of gene expression in the discrimination of AIA from ATA and healthy subjects, we selected candidate genes that satisfied the criteria of *p* < 0.05 and exhibiting change in expression greater than twofold difference between the two chosen groups. For microarray analysis, background-corrected values for each probe on oligonucleotide array were extracted using MeV software (TM4, Boston, MA).

#### qPCR and immunoblotting analysis

For qPCR and immunoblotting results, the distribution of the log_2_data and the equality of variances were checked by Shapiro-Wilk and Levene’s tests, respectively. The results were presented as mean ± SEM when data in groups were normally distributed; differences between groups were examined for statistical significance by ANOVA with the appropriate post-hoc test. If Kruskal - Wallis test (with multiple comparison) - non-parametric equivalent of ANOVA was used, the results were presented as median ± range.

*P value* < 0.05 was considered as statistically significant. The data from the study was analyzed utilizing STATISTICA software package (Statsoft, Tulsa, OK).

#### Power analysis

Sample size was calculated based on the number of aspirin sensitive patients counted per total population of Poland (6). Based on Daniel formula for calculating sample size (29), this gave a calculated AIA sample size approximately 9 patients. However, a higher number was targeted in qPCR in order to account for possible exclusions, dropouts and the need to carry out subgroup analysis.

## Results

### Comparison of gene expression profiles between AIA *versus* ATA and AIA *versus* healthy volunteers

**Fig. 1:**
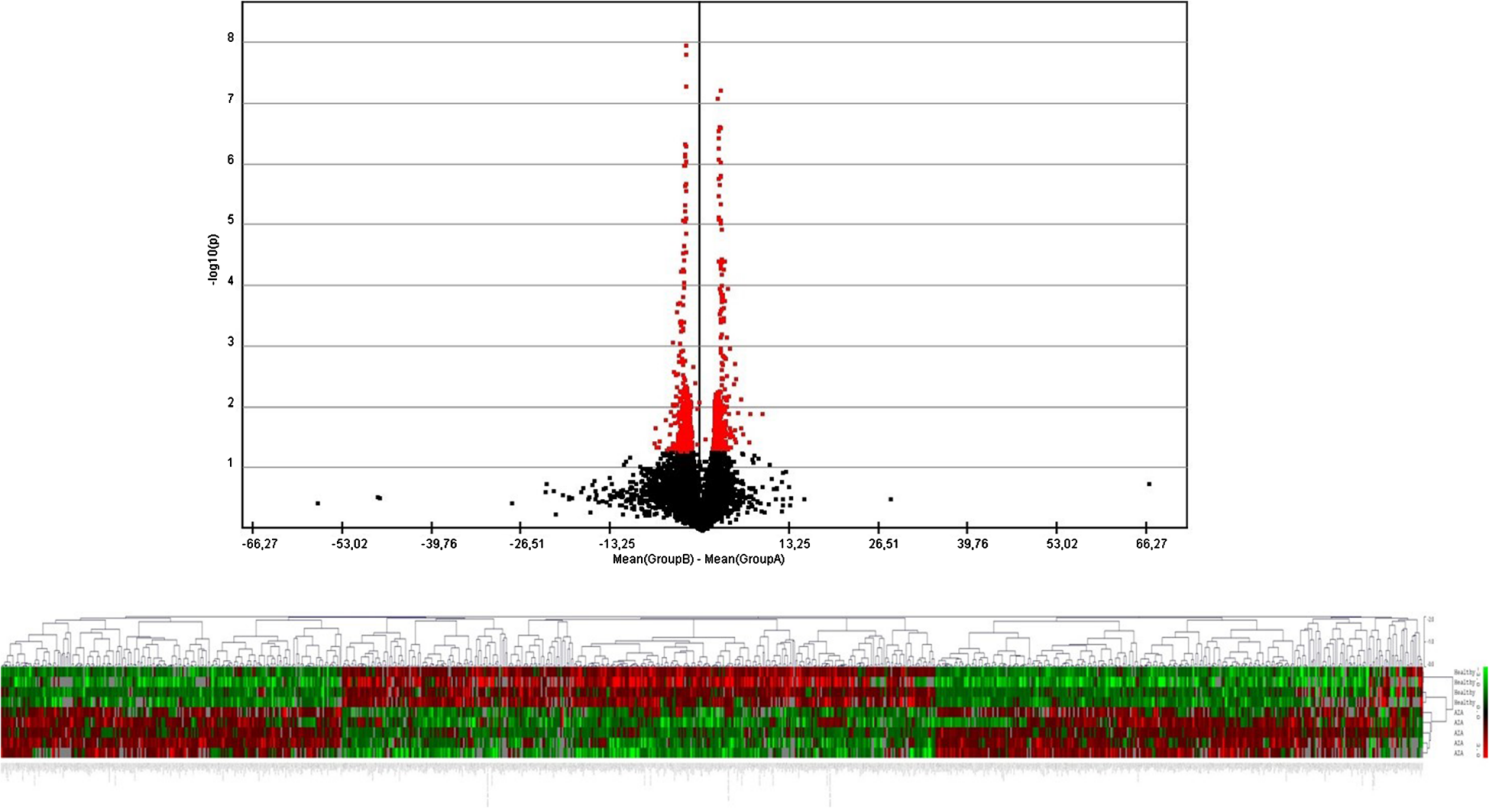
Volcano plot and result of hierarchical clustering for *p* < 0.05 obtained for aspirin-induced asthma and aspirin-tolerant asthma patients

**Fig. 2:**
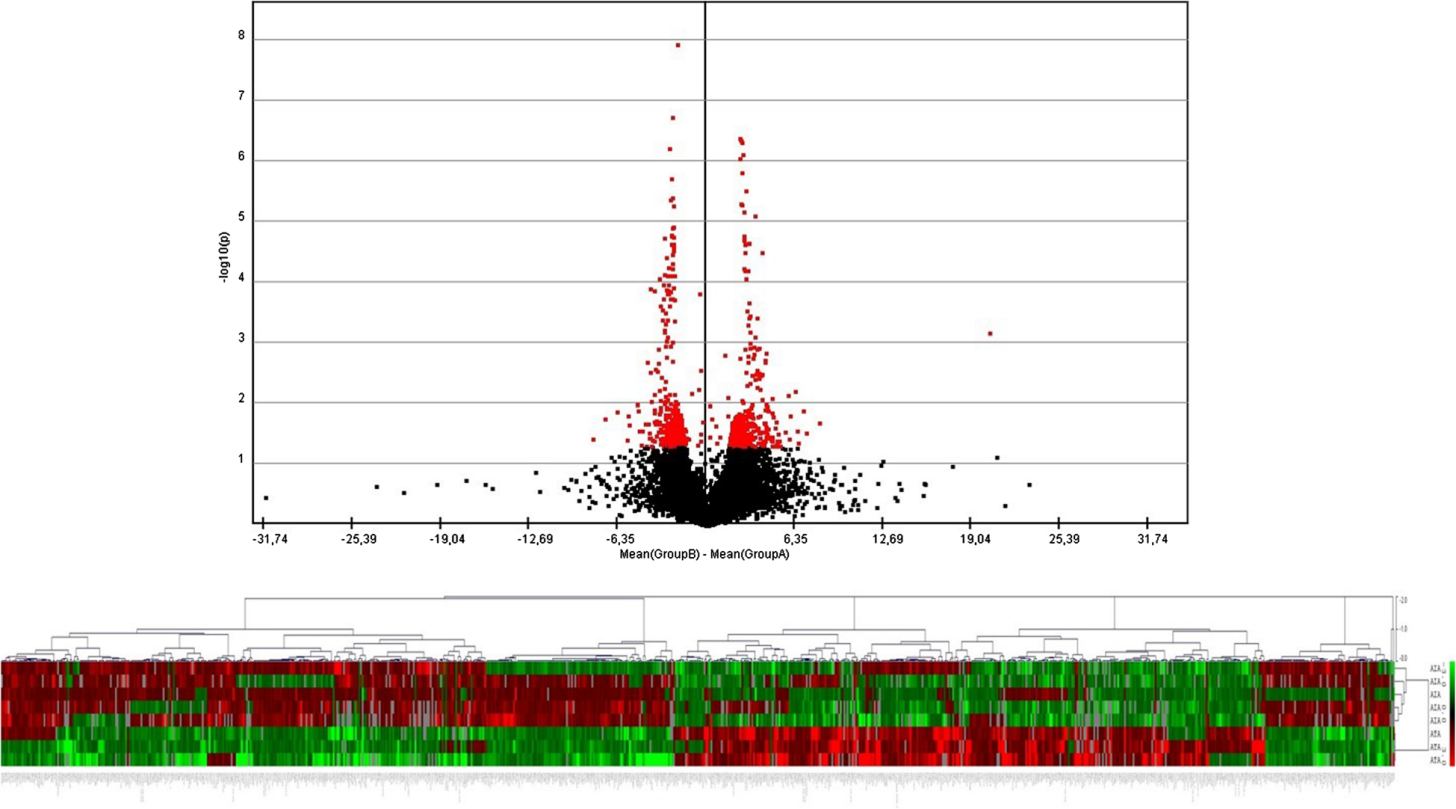
Volcano plot and result of hierarchical clustering for *p* < 0.05 obtained for healthy subjects and aspirin-induced asthma patients

**Fig. 3:**
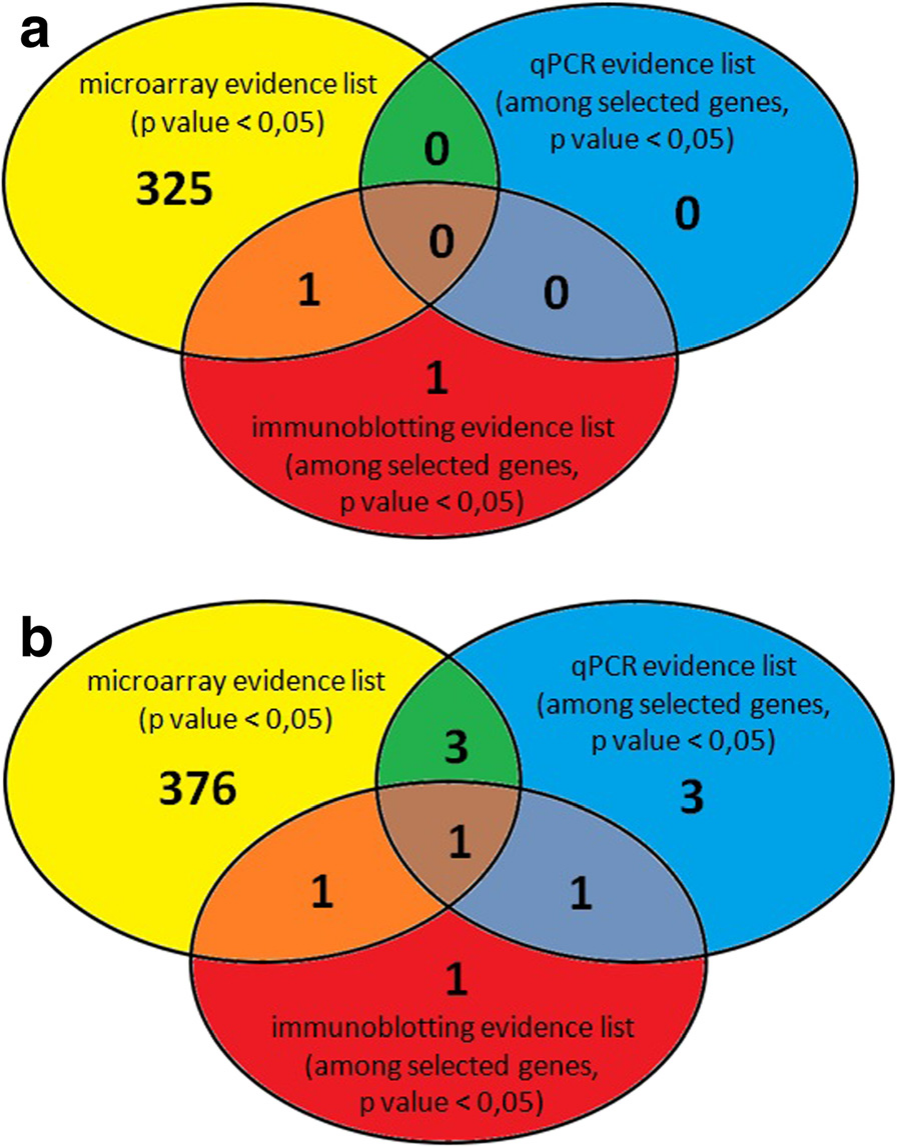
Venn diagram of overlap between gene numbers predicted from microarray, qPCR and immunoblotting methods for AIA *versus* ATA (**a**) and AIA *versus* HV (**b**)

**Fig. 4:**
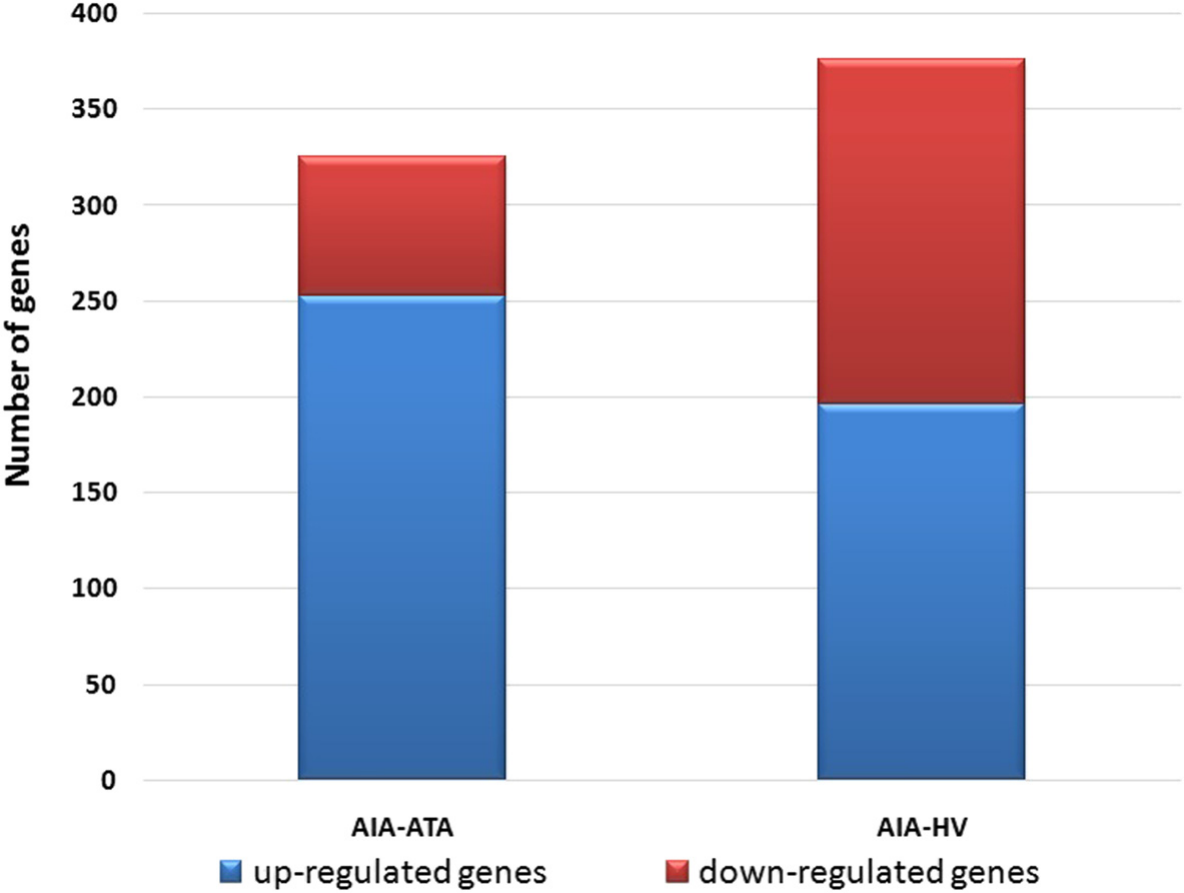
Number of genes with differential expression between groups with up-regulated genes being predominant on AIA *versus* ATA and AIA *versus* healthy volunteers. PBMCs were separated from blood of three groups of subjects - 11 AIA, 7 ATA and 15 HV and stimulated by either lysine aspirin or lysine as a control. The gene expression presented was analyzed utilizing microarray technique

The gene expression microarray consisting of 30.255 featured oligonucleotide probes to cDNA samples obtained from AIA (*n* = 5), ATA (*n* = 3) and healthy volunteers (*n* = 4) was applied. To evaluate the overall difference in gene expression levels in PBMCs among AIA, ATA and healthy volunteers, we calculated the gene expression level using a volcano plot (Figs. 1 and 2). Volcano plot against fold change values for each gene revealed that the expression levels were slightly different between AIA *versus* ATA and AIA *versus* healthy subjects. We identified genes that showed 325 significantly different expression between AIA *vs.* ATA (253 genes that showed a significant increase in gene expression and 72 genes that showed a significant decrease) and 376 genes with significantly changed expression between AIA *versus* healthy volunteers – 196 genes turned out to be significantly increased and 180 genes with statistically significant decreased expression (Figs. 3 and 4).

**Fig. 5:**
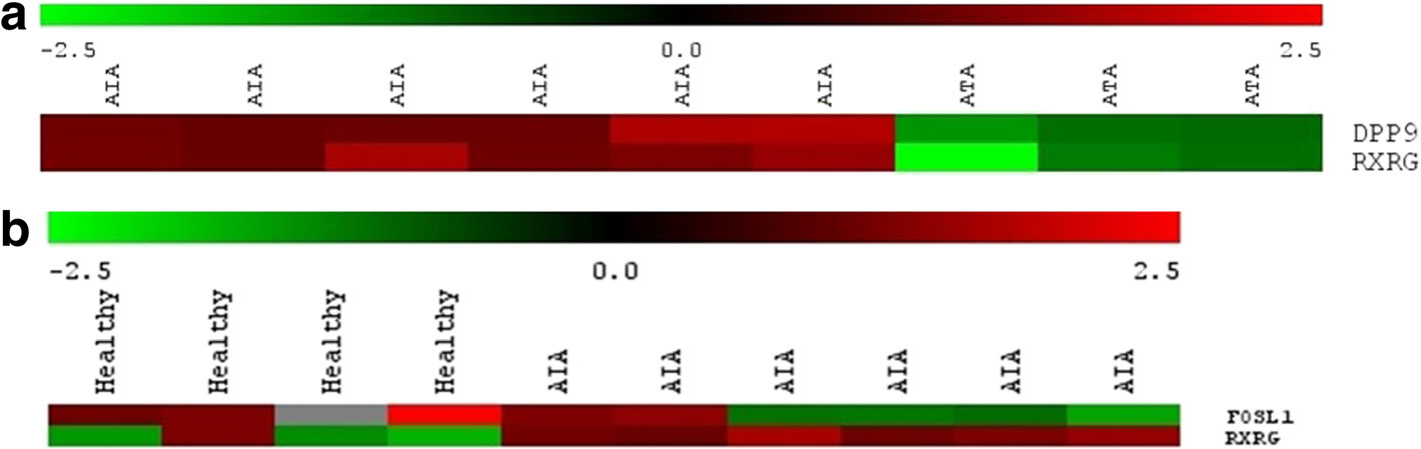
Heat map of selected genes of AIA and ATA subjects (*p* < 0.05) (**a**), as well as healthy controls and AIA subjects (*p* < 0.05) (**b**)

For the next step of analysis, we selected genes DPP9, RXRG and FOSL1 with a *p value* of <0.05 and mean difference in fold change value >2 between the two chosen groups (Fig. 5). Differences in gene expression obtained in whole genome scan using cDNA microarrays was shown in Table 4. The role of selected genes in inflammation or asthma had been confirmed in literature before. The upregulated and downregulated genes were perfectly classified by the hierarchical clustering method.

**Table 4:** Differences in gene expression obtained in *whole genome scan* using cDNA microarrays

No	Gene symbol	AIA mean	ATA mean	*p* value	FDR
1	*DPP9*	1,29	- 1,20	1,23 · 10^−5^	0,009
2	*RXRG*	1,28	- 1,80	2,61 · 10^−4^	0,089
No	Gene symbol	AIA mean	HV mean	p value	FDR
3	*FOSL1*	−0,39	2,42	0,044	1,189

### Verification of gene expression with quantitative measurement of mRNA using qPCR

We validated three previously selected genes: *DPP9, RXRG* and *FOSL1* using qPCR to measure their mRNA levels in PBMCs obtained from AIA (*n* = 11), ATA (*n* = 7) and healthy volunteers (*n* = 15). Therefore qPCR was analyzed for original microarray patients’ group and additional patients were added to have a confirmatory cohort.

Quantification of mRNA levels was performed by measuring the amount of *DPP9, RXRG* and *FOSL1* qPCR product after correcting for amount of *GAPDH.* Additionally, qPCR analysis included validation of such genes as *ALOX5, ALOX15, BMP2, CNPY3, CNPY3, CSF1, CXCL11, DOCK9, ERAS, GAB3, MARVELD1, PARVG, TLR7* and *TRIP6* – markers of inflammation, formerly described in literature.

### mRNA expression of selected genes

**Fig. 6:**
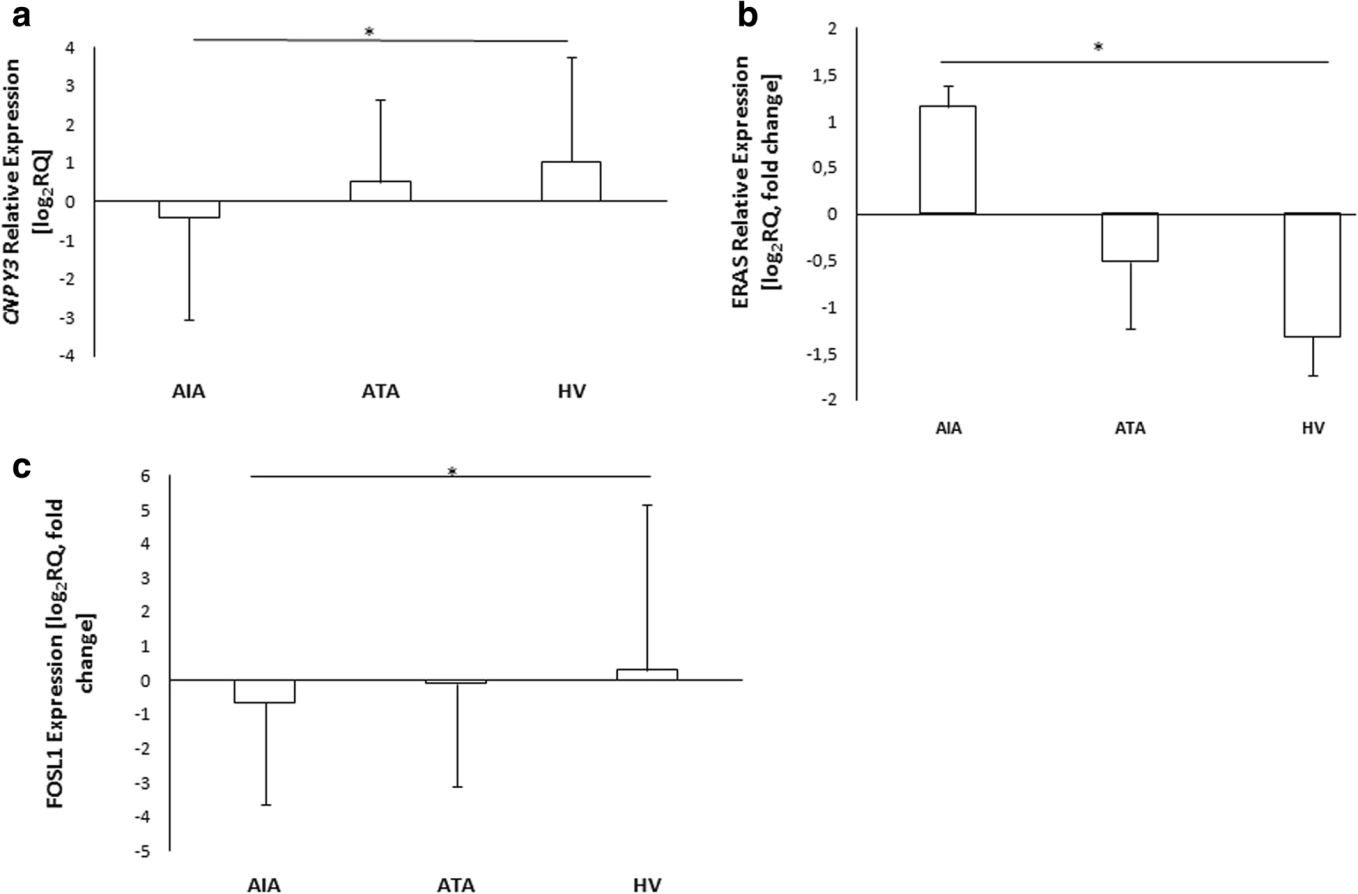
mRNA expression levels of *CNPY3* **a** *, FOSL1* **b** *and ERAS* **c** genes in PBMCs measured by qPCR between AIA (*n* = 11), ATA (*n* = 7) and healthy volunteers (*n* = 15). PBMCs were stimulated by lysine-aspirin or lysine as a control. The gene expression presented was analyzed utilizing Real-Time PCR. * statistically significant (*p* < 0.05)

mRNA expression of *CNPY3* in PBMCs in AIA was significantly lower (-0.41 ± 2.67) than in healthy group (1.04 ± 2.69), (*p* = 0.02). However, expression of the mRNA of *CNPY3* in PBMCs was not significantly different between AIA and ATA (0.52 ± 2.15), (*p* = 0.08) and ATA *vs.* healthy group (*p* = 1.00); (Fig. 6a).

mRNA expression of *ERAS* in PBMCs in AIA was significantly higher (1.15 ± 0.23) than in healthy group (-1.32 ± 0.41), (*p* = 0.03). While, expression of the mRNA of *ERAS* in PBMCs was not significantly different between AIA and ATA (-0.52 ± 0.72), (*p* = 0.18) and ATA *vs.* healthy group (*p* = 0.65); (Fig. 6b).

mRNA expression of *FOSL1* in PBMCs in AIA was significantly lower (-0.66 ± 2.97) than in healthy group (0.31 ± 4.83), (*p* = 0.02). However, *FOSL1* mRNA expression in PBMCs was not significantly changed between AIA and ATA (-0.09 ± 3.03), (*p* = 0.54) and ATA *vs.* healthy group (*p* = 0.78); (Fig. 6c).

**Fig. 7:**
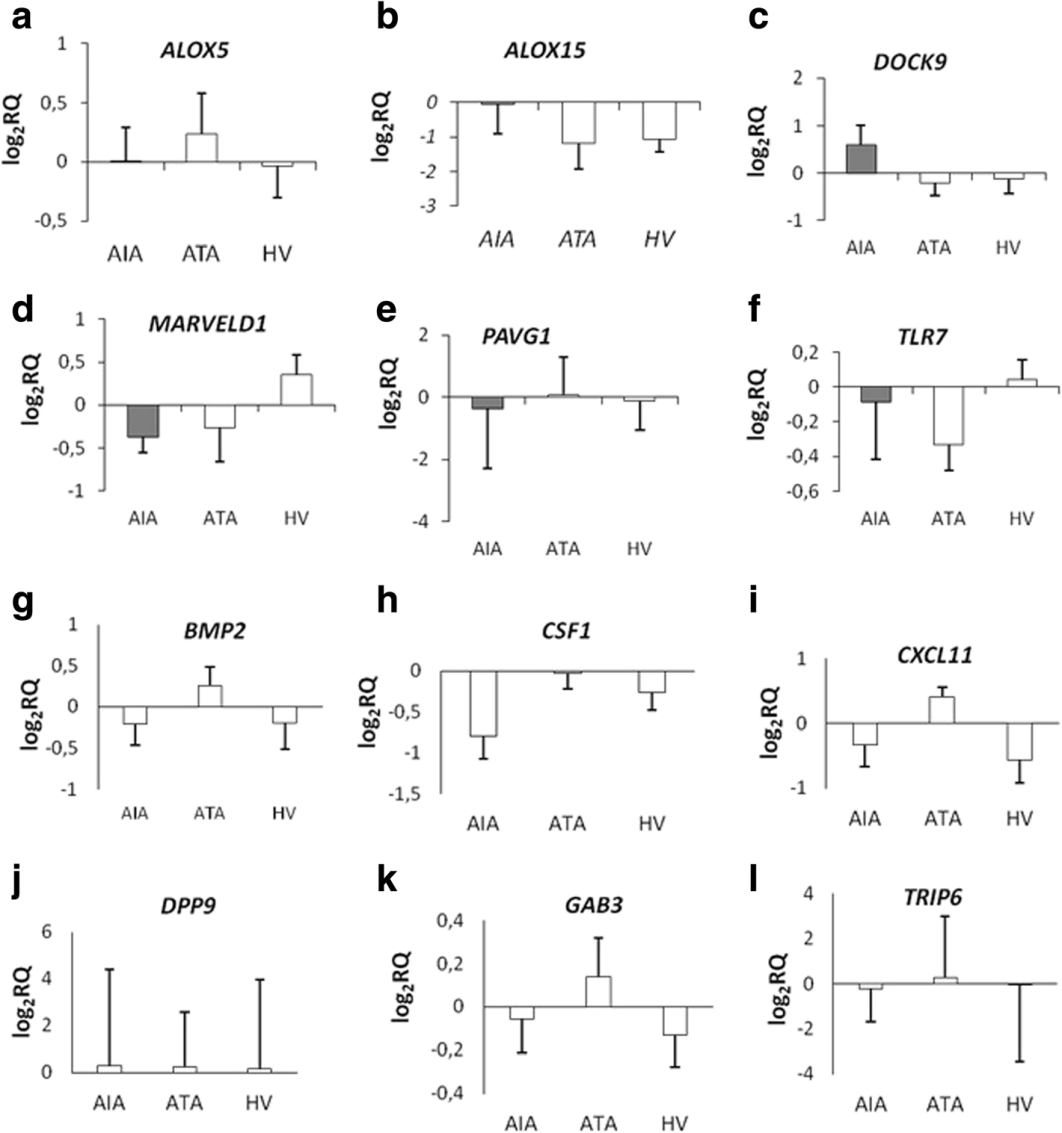
Box plot for mRNA expression levels of *ALOX5* **a** *, ALOX15* **b** *, DOCK9* **c** *, MARVELD1* **d** *, PARVG* **e** *, TLR7* **f** *BMP2* **g**, *CSF1* **h**, *CXCL11* **i**, *DPP9* **j**, *GAB3* **k**, and *TRIP6* **l** genes in PBMCs measured by qPCR between AIA (*n* = 11), ATA (*n* = 7) and healthy volunteers (*n* = 15). PBMCs were stimulated by lysine-aspirin or lysine as a control. The gene expression presented was analyzed utilizing Real-Time PCR

mRNA expression of the following genes in PBMCs was not significantly changed after aspirin-lysine and lysine incubation:

ALOX5 - AIA (0.01 ± 0.28) *vs.* ATA (0.23 ± 0.35), (*p* = 0.894) and healthy volunteers (-0.04 ± 0.27); (*p* = 0,99) (Fig. 7a), *ALOX15* - AIA (-0.05 ± 0.86) *vs.* ATA (-1.18 ± 0.75), (*p* = 0.5) and healthy volunteers (-1.07 ± 0.36), (*p* = 0.52); (Fig. 7b).

*DOCK9 -* AIA (0.59 ± 0.42) *vs.* ATA (-0.21 ± 0.26), (*p* = 0.39) and healthy volunteers (-0.12 ± 0.31), (*p* = 0.42); (Fig. 7c**)**, *MARVELD1* - AIA (-0.37 ± 0.18) *vs.* ATA (-0.27 ± 0.4), (*p* = 0.97) and healthy volunteers (0.35 ± 0.23), (*p* = 0.15); (Fig. 7d).

*PARVG* - AIA (-0.35 ± 1.93) *vs.* ATA (0.07 ± 1.2), (*p* = 0.14) and healthy volunteers (-0.12 ± 0.92), (*p* = 0.77); (Fig. 7e), and *TLR7* - AIA (-0.09 ± 0.33) *vs.* ATA (-0.33 ± 0.15), (*p* = 0.8) and healthy volunteers (0.04 ± 0.11), (*p* = 0.9); (Fig. 7f).

Moreover, no statistically significant changes were observed in the following genes mRNA expression:

*BMP2* - AIA (-0.22 ± 0.25) *vs.* ATA (0.26 ± 0.23), (*p* = 0.72) and healthy volunteers (-0.20 ± 0.31), (*p* = 1); (Fig. 7g), *CSF1* - AIA (-0.8 ± 0.28) *vs.* ATA (-0.03 ± 0.18), (*p* = 0.23) and healthy volunteers (-0.27 ± 0.21), (*p* = 0.31); (Fig. 7h).

*CXCL11* - AIA (-0.33 ± 0.33) *vs.* ATA (0.4 ± 0.15), (*p* = 0.41) and healthy volunteers (-0.57 ± 0.35), (*p* = 0.87); (Fig. 7i), *DPP9* - AIA (0.28 ± 4.12) *vs.* ATA (0.24 ± 2.34), (*p* = 1) and healthy volunteers (0.16 ± 3.8), (*p* = 1.0); (Fig. 7j).

mRNA expression of *GAB3* in PBMCs was also not significantly changed between AIA (-0.06 ± 0.13) *versus* ATA (0.14 ± 0.25), (*p* = 0.75) and healthy patients (-0.13 ± 0.12), (*p* = 0.95); (Fig. 7k), similarly, *TRIP6* in PBMCs - AIA (-0.25 ± 1.46) *vs.* ATA (0.26 ± 2.73), (*p* = 1) and healthy patients (-0.03 ± 3.43), (*p* = 1); **(**Fig. 7l).

### Verification of gene expression with quantitative measurement of protein using immunoblotting analysis

Comparison of protein expression encoded by selected genes between AIA (*n* = 6), ATA (*n* = 6) and healthy volunteers (*n* = 7) groups was done by means of immunoblotting. Quantification of ALOX5, ALOX15, BMP2, CNPY3, CSF1, CXCL11, DOCK9, DPP9, ERAS, FOSL1, GAB3, MARVELD1, PARVG, TLR7, TRIP6 protein levels was performed by measuring the protein product amount after stimulation of PBMCs with lysine aspirin and correcting for obtained protein amount after stimulation of PBMCs with lysine. Further, quantification of protein levels on the basis of obtained bands after stimulation with lysine aspirin and lysine was analyzed on the same gel for each patients. Sharp bands for BMP2, CNPY3, CSF1, CXCL11, DPP9 ERAS, FOSL1, GAB3 and TRIP6 appeared in the expected positions. In the case of other selected genes, no or faint bands (weak signal) were obtained.

### Selected proteins expression

**Fig. 8:**
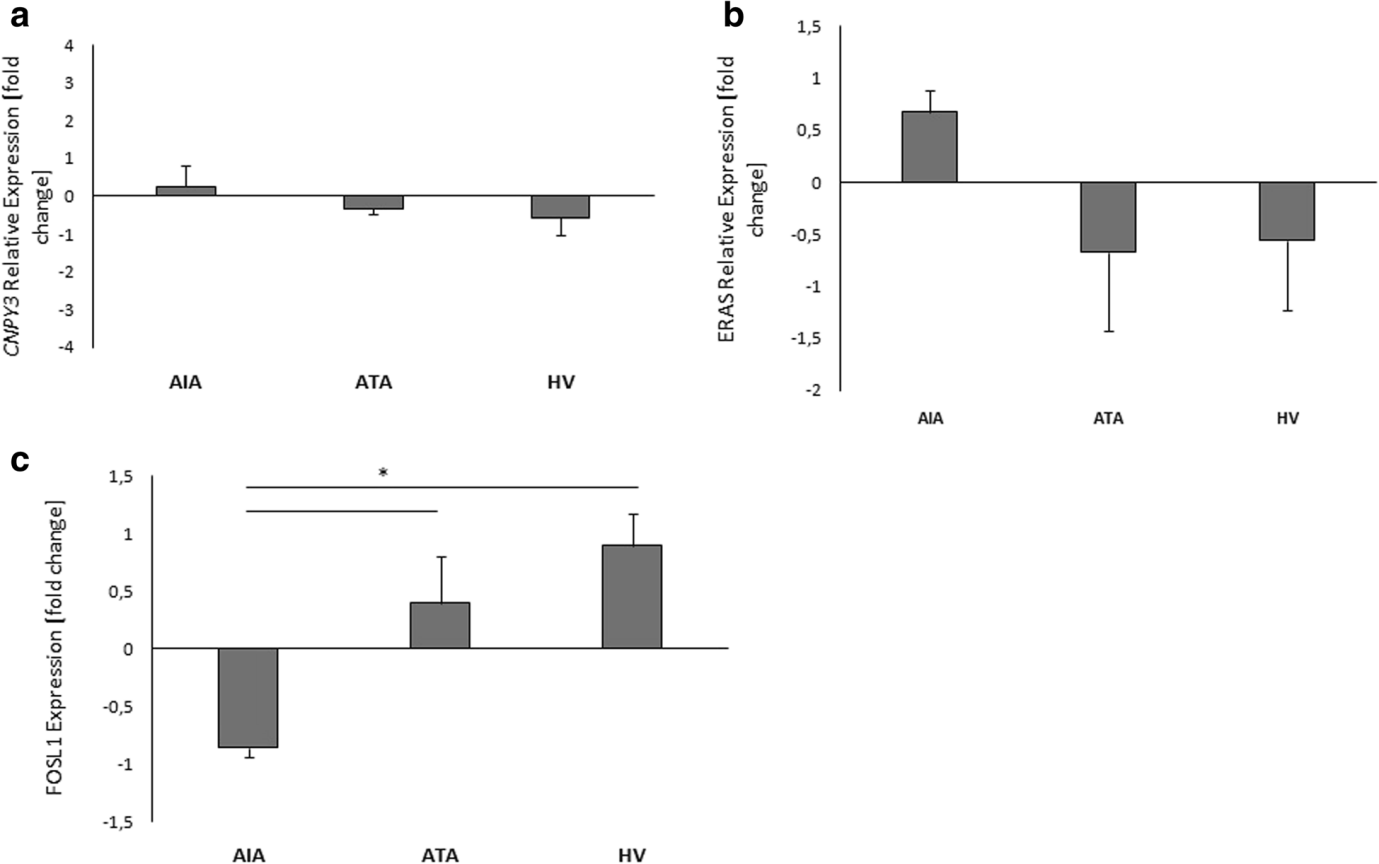
Protein expression levels of *CNPY3* **a** *, FOSL1* **b** *and ERAS* **c** genes in PBMCs measured by qPCR between AIA (*n* = 11), ATA (*n* = 7) and healthy volunteers (*n* = 15). PBMCs were stimulated by lysine-aspirin or lysine as a control. Protein data are presented as the fold change of optical density (OD) compared with the lysine – treated cells. *statistically significant (*p* < 0.05)

CNPY3 protein expression in PBMCs was not significantly changed between AIA (0.26 ± 0.55) *vs.* ATA (-0.33 ± 0.16), (*p* = 0.58) and healthy patients (-0.56 ± 0.47), (*p* = 0.41); (Fig. 8a), same as ERAS protein expression - AIA (0.68 ± 0.21) *vs.* ATA (-0.67 ± 0.75), (*p* = 0.37) and healthy patients (-0.56 ± 0.67), (*p* = 0.38); (Fig. 8b).

**Fig. 9:**
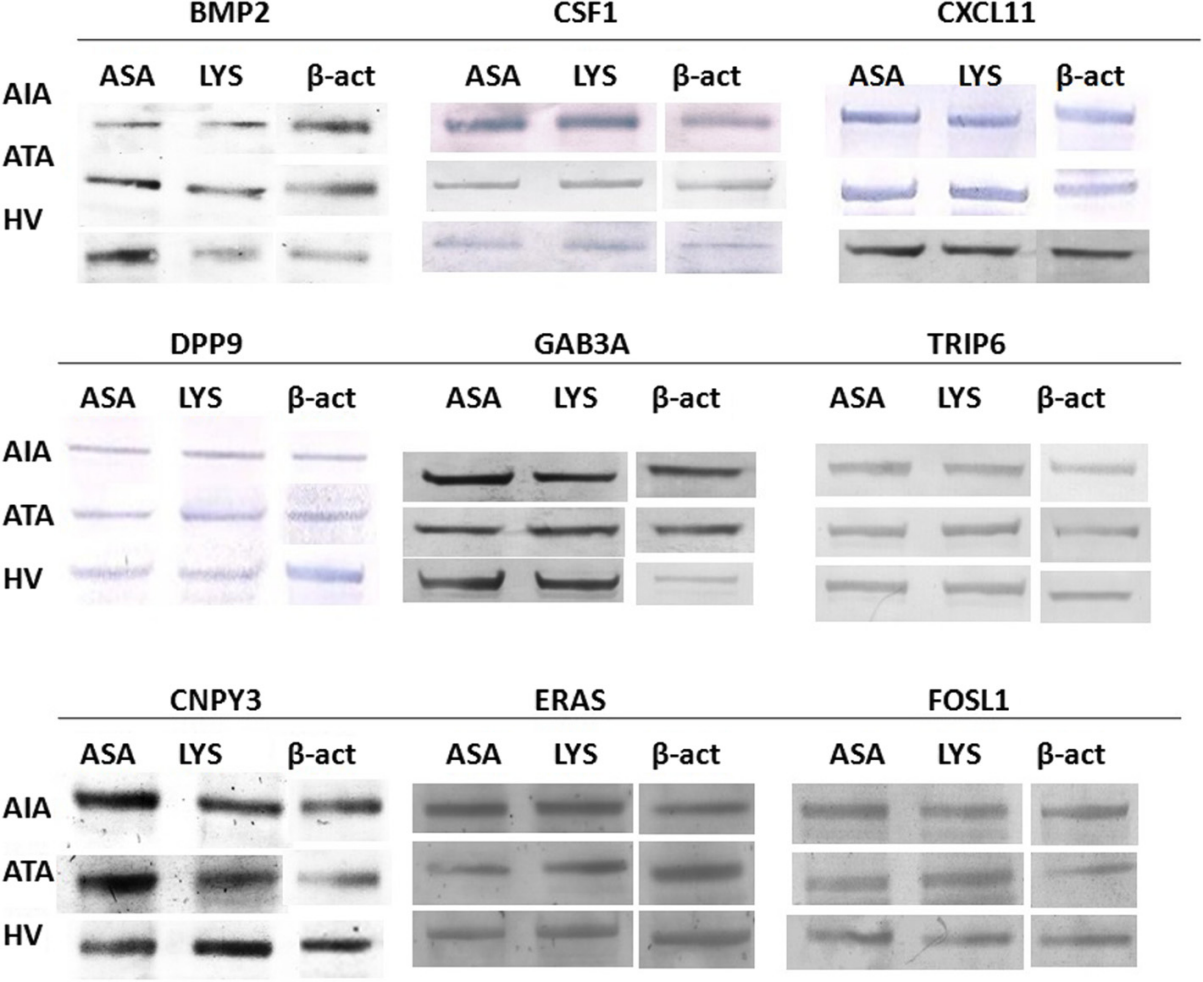
Protein expression measured by immunoblotting between AIA, ATA and healthy volunteers. The blots shown are a representative of at least six separate experiments that gave similar results

Protein expression of *FOSL1* in PBMCs in AIA was significantly lower (-0.86 ± 0.08) in comparison to ATA (0.39 ± 0.42), (*p* = 0.046) and to healthy subjects (0.9 ± 0.27), (*p* = 0.007). However, *FOSL1* protein expression in PBMCs was not significantly changed between ATA *vs.* healthy group (*p* = 0.487); **(**Fig. 8c). Immunobloting results are shown in the Fig. 9.

**Fig. 10:**
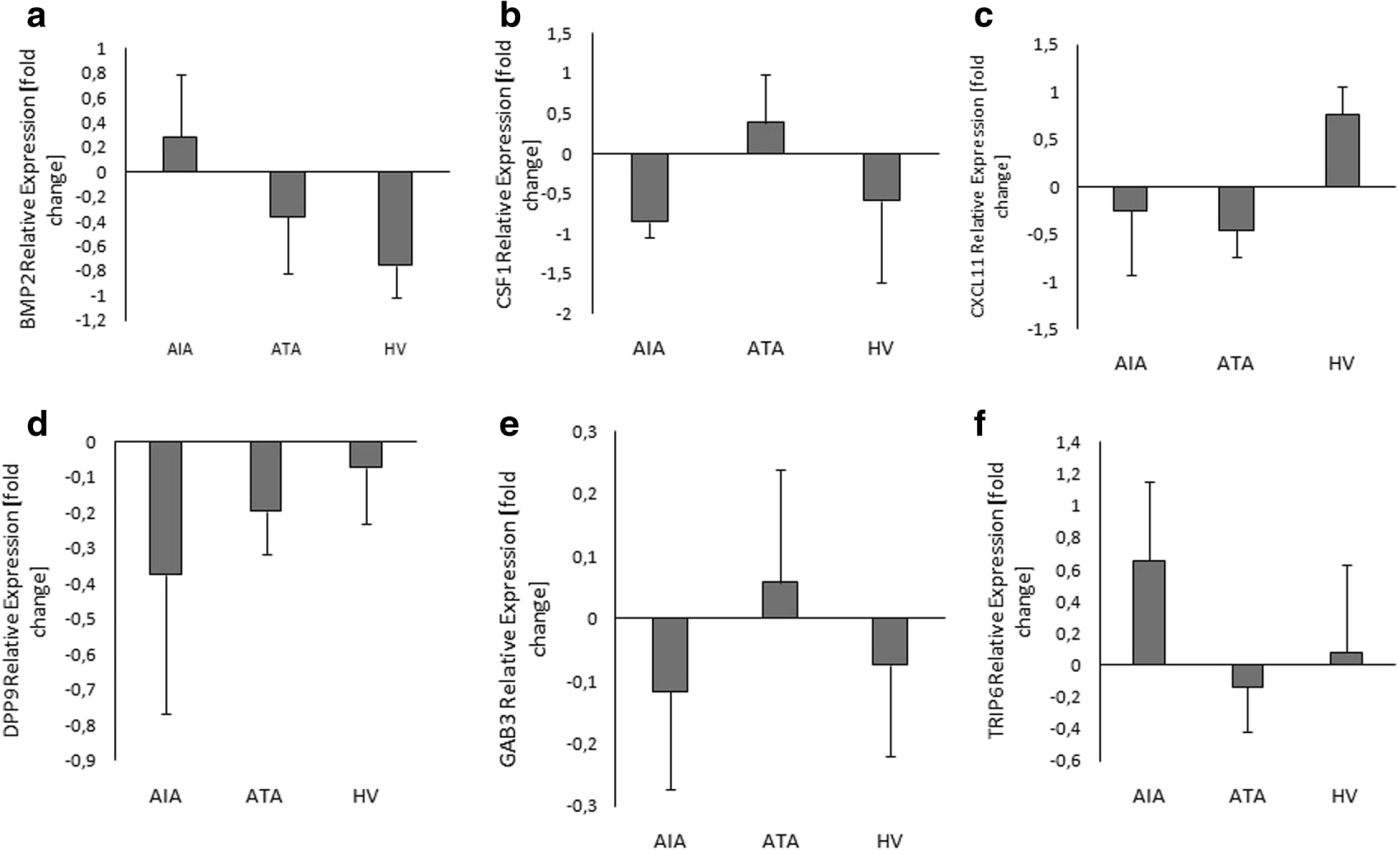
Box plot for protein expression levels of *BMP2* **a**, *CSF1* **b**, *CXCL11* **c**, *DPP9* **d**, *GAB3* **e** *and TRIP6* **f** genes in PBMCs measured by qPCR between AIA (*n* = 11), ATA (*n* = 7) and healthy volunteers (*n* = 15). Data are presented as the fold change of optical density (OD) compared with the lysine – treated cells

Changes in expression of the other proteins were not statistically significant: BMP2 protein expression in PBMCs was not significantly changed between AIA (0.28 ± 0.5) *vs.* ATA (-0.36 ± 0.46), (*p* = 0.58) and healthy patients (-0.76 ± 0.26), (*p* = 0.2); (Fig. 10a), the same as CSF1 protein - AIA (-0.85 ± 0.2) vs*.* ATA (0.39 ± 0.6), (*p* = 0.34) and healthy patients (-0.59 ± 1.0), (*p* = 0.9); (Fig. 10b), and CXCL11 - AIA (-0.25 ± 0.67) *vs.* ATA (-0.46 ± 0.28), (*p* = 0.94) and healthy patients (0.77 ± 0.28), (*p* = 0.28); (Fig. 10c).

DPP9 protein expression in PBMCs was not significantly changed between AIA (-0.37 ± 0.4) *vs.* ATA (-0.2 ± 0.12), (*p* = 0.91) and healthy patients (-0.07 ± 0.16), (*p* = 0.76); (Fig. 10d), the same as GAB3 protein expression - AIA (-0.12 ± 0.16) *vs.* ATA (0.06 ± 0.18), (*p* = 0.74) and healthy patients (-0.07 ± 0.14), (*p* = 0.98); (Fig. 10e**)**, and TRIP6 protein - AIA (0.66 ± 0.5) vs*.* ATA (-0.14 ± 0.28), (*p* = 0.54) and healthy patients (0.08 ± 0.55), (*p* = 0.72); **(**Fig. 10f).

## Discussion

Considering the genetic background of AIA, more than 100 genetic association studies have attempted to discover the numerous genetic variants related to development of AIA. However, the majority of these results have not been replicated in other, independent studies. Moreover, to the best of our knowledge, two published papers based on both microarray study and qPCR confirmation reveal the involvement of individual genes in the pathogenesis of AIA. However neither of which were also confirmed in other studies and population.

The first, whole-genome study [36] demonstrated that galactin-10 mRNA is overexpressed in peripheral blood cells of AIA compared to ATA patients and controls. Galactin-10 had been previously implicated in mucosal inflammatory processes including cell adhesion [37], chemoattraction [38] and cell activation [39]. Whereas, the second study [40] showed two genes – *CNKSR3* and *SPTBN2* which expression in PBMCs differentiates between AIA and ATA, but neither *CNKSR3* nor *SPTBN2* has described relationship with asthma and aspirin.

As in previous whole genome studies, the main aim of our investigation was to compare the AIA genetic profile against ATA and HV in PBMCs by microarray studies and then confirm it on protein level. The verification on two molecular levels was necessary because mRNA levels cannot be utilized as surrogates for corresponding protein levels. Although RNAs are primordial molecules, proteins are the molecules of life and it is estimated that only less than 40 % of cellular protein levels can be predicted from mRNA measurements [41]. The most known, presumable reasons for the poor correlations reported in literature between the level of mRNA and protein are: (a) many complicated and varied post-transcriptional mechanisms involved in turning mRNA into protein – the cell can control the levels of gene at transcriptional level and/or translational level [42]; (b) difference in half-lives of proteins as the result of varied protein synthesis and degradation depending on a number of different conditions; (c) significant amount of error in mRNA/protein studies [43, 44]. Intriguingly, genes with certain combinations of mRNA and protein half-lives share common functions, indicating that they evolved under similar constraints such as abrupt respond to stimulus [41, 45–47]. Most mRNAs and especially proteins are stable unless genes need to respond quickly to a stimulus [41]. However, measurements performed at mRNA and protein levels are complementary and both are necessary for a complete understanding how the cell works [48].

On the basis of obtained results, we identified three genes whose expression profiles significantly differed between AIA *vs.* ATA and/or AIA *vs.* healthy subjects in PBMCs of Caucasian population. We demonstrated significant decrease in expression of *FOSL1* (encoding FRA1) at either mRNA or protein level in patients diagnosed with AIA in comparison to ATA and controls. *FOSL1* is a part of AP-1 – transcription factor that regulates target gene expression in response to various pro-oxidants, inflammatory cytokines including TGFβ1 [49, 50], environmental toxicants, carcinogens and pathogens. These gene products mediate oxidative stress and inflammatory responses, as well as cell growth and tumorgenesis [51]. Additionally, TGF-β1 promoter (509C/T) polymorphism has been reported to contribute to the development of AIA with rhinosinusitis by increasing TGF-β production in the nasal mucosa and/or polyp tissues of patients with AIA [52]. Tang et al. showed that aspirin-treated bone marrow cells have significantly improved immunomodulatory function, as indicated by upregulation of regulatory T cells and downregulation of Th17 cells via, *inter alia* TGF-β1 pathway [53].

Moreover, *FOSL1* regulates the expression of genes controlling tissue/cell remodeling, mainly at transcriptional level [54–56]. Rajasekaran et al. [57] have recently shown that FRA1^-/-^ mice are more susceptible than wild-type mice to bleomycin - induced fibrosis, suggesting that this transcriptional factor is involved in pulmonary protection. To emphasize this hypothesis, downregulation of *FOSL1* was also observed in malignant human bronchial epithelial cells [55] and non-small-cell lung cancer [58] compared to normal bronchial epithelium.

Comparison of genetic profile between AIA and healthy controls has also demonstrated significantly increased expression of *ERAS* in AIA. Actually the role of this gene is restricted to the tumor - like growth properties of embryonic stem cells [59] and chemotherapy resistance [60]. However, *ERAS* belongs to GTPase Ras protein family engaged in airway smooth muscle growth and bronchoconstriction of airways in response to stimuli [61]. Among all proteins that belong to Ras superfamily, Rho kinase has emerged as a potential target for the treatment of airway hyperresponsiveness in asthma [62]. Additionally, arachidonic acid (AA) can activate Rho kinase by binding to the C-terminal part of the coiled-coil domain of Rho kinase, which acts as an auto-inhibitor domain [63–65]. Rho kinase may also be involved in eotaxin and cytokine (IL-5, IL-13) production [66] and in secretion of matrix metalloproteinase – 9 (MMP-9), tightly associated with fibrosis in asthma and chronic obstructive pulmonary disease (COPD) [67, 68]. It is worth mentioning, as the extent of Ras activation in T cells appears to drive Th2 dependent eosinophilic airway inflammation and allergen-induced airway hyperresponsiveness [69]. Much evidence indicates also that Ras GTPases appear to regulate reactive oxygen species (ROS) production and oxidants function as effector molecules for the small GTPases [70–73]. Rac1 has been demonstrated to act upstream of AA – metabolizing enzymes, such as PLA_2_ [74, 75], 5-LOX [76–78] and COX-2 [79] and thus some reports show that AA metabolism modulates NADPH oxidase and mitochondrial ROS production [80].

The misregulation of the redox signaling of Ras with its downstream cascades also has been linked to various disorders linked with immune system [81]. According to Wells et al. [82], Ras-dependent Raf-MEK1/2-ERK1/2 pathway takes part in postnatal modulation of a host’s defenses and the inflammation of T lymphocytes. In a mouse allergic asthma model, the activation of Ras in T cells controls the development of Th2-dependent eosinophilic airway inflammation and airway hyperresponsiveness. Specific inhibitors focusing on Ras-mediated signaling pathways would be thus helpful in treatment approach of asthma [69].

Although ERAS was one of the genes indicating association with aspirin-induced asthma in our study, there are only single data supporting its role. Nevertheless, recently, Park et al. [83] have shown a strong association between the SNPs (14444 T > G and 41170 C > G) within RAB1A (Ras protein subfamily member) and the aspirin-induced decrease in FEV_1_. The authors indicate also, that genetic alteration of the member RAS oncogene family may be related to the development of asthma and ASA hypersensitivity through the modulation of intracellular protein trafficking.

Multiple points of overproduction or underproduction of critical inflammatory mediators may be determined by metabolism through the Ras family GTPase pathway. The release of specific granules from platelets, eosinophils, and neutrophils depends on the phosphorylation of the Ras family proteins [81], but detailed mechanism associated with aspirin-induced asthma needs to be evaluated.

Significantly reduced expression of *CNPY3* at mRNA level in AIA in comparison to healthy controls may indicate a profound defect in stimulus responsiveness. CNPY3 is an endoplasmic reticulum - resident chaperone that is required for maturation/ glucosylation and surface trafficking of TLR4 [84]. Activated TLR4 can directly or indirectly affect the function of regulatory T cells, thus influencing the Th1/Th2 imbalance and reducing inflammatory responses [85–87].

It is well known, that TLR4 is important component in the innate immune response to lipopolysaccharide (LPS) of gram-negative bacteria and the fusion protein of respiratory syncytial virus (RSV) [88]. Therefore, CNPY3 knockdown led to significant defect in RSV and LPS responsiveness and limit innate immune responses [84, 89]. By contrast, patients with AIA much more frequently suffer from virus infection [90] and RSV is probably one of the trigger predisposing to aspirin hypersensitivity [91].

TLR4 is activated following binding of LPS, and a series of downstream phosphorylation and dephosphorylation events eventually leads to the activation of transcription factors that regulate inflammatory factors including interferon, tumor necrosis factor; it also induces antigen-presenting cell maturation and promotes a Th0 to Th1 shift [85, 92]. According to Steinke et al. [93], high levels of mentioned IFN-γ distinguish AERD (aspirin-exacerbated respiratory disease) from aspirin tolerant asthma and underlie the robust constitutive and aspirin-induced secretion of CysLTs that characterize this disorder, as AERD is associated with eosinophils maturing locally in a high interferon (IFN)-γ.

To better understand the contribution of TLR4 to aspirin-induced asthma pathogenesis, additional studies are needed to determine the contribution of CNPY3 in aspitin-induced asthma.

Our data also demonstrate that similar microarray scores for different genes do not necessarily mean that similar qPCR scores was obtained. This finding presumably reflects the different hybridization kinetics of the probe sets for each gene. Furthermore, varied priming methods and increased distance between the location of the PCR primers and microarray probes on a given gene can also affect the results of qPCR and microarray experiments. In addition, data normalization fundamentally differs between microarray analysis and qPCR, the former requiring global normalization, while the latter generally utilizes the expression of one reference gene against which all other gene expression is calibrated. Therefore, on the basis of the qPCR data that we obtained, it is generally not feasible to predict the true expression level of one gene based on the microarray expression score of another.

## Conclusions

To sum up, altered expression of three genes: *ERAS*, *CNPY3* and *FOSL1* have been reported at mRNA level in PBMCs of Caucasian aspirin-sensitive asthmatics as opposed to healthy volunteers. In the case of FOSL1, this difference was also confirmed at protein level, both - between AIA *vs.* ATA and AIA *vs.* HV. To our knowledge, this is the first whole-genome study for AIA that points out the positive association between ERAS, CNPY3, FOSL1 and NSAIDs metabolism. However, some previous studies have indicated participation of these genes in pathways significant for pathomechanism of AIA resulting in tissue/cell remodeling and airway hyperresponsiveness. Although our study included small number of patients, it allowed to perform statistical analysis. Undoubtedly, further studies in a larger number of cases and of other ethnicity are necessary to establish an exact functional link among the detected alternations in expression of CNPY3, ERAS and FOSL1 with pathology of AIA.

## Additional file

Additional file 1.
**Western Blot protocol.** (PDF 176 kb)

## Abbreviations

15-HETE: 15 – hydroxyeicosatetranoic acid
15-LOX: 15 – lipooxygenase
5-LOX: 5-lipoxygenase
AA: arachidonic acid
AERD: aspirin-exacerbated respiratory disease
AIA: aspirin-induced asthma
ALOX15: arachidonate 15-lipoxygenase
ALOX5: arachidonate 5-lipoxygenase
ASA: aspirin
ATA: aspirin tolerant asthma
BMP2: bone morphogenetic protein 2
CNKSR3: CNKSR family member 3
CNPY3: canopy FGF signaling regulator
COX: cyclooxygenase
CSF1: colony stimulating factor 1 (macrophage)
CXCL11: chemokine (C-X-C motif) ligand 11
cysLTR1: type 1 receptor for cysteinyl leukotriens
DOCK9: dedicator of cytokinesis 9
DPP9: dipeptidyl-peptidase 9
ERAS: ES cell expressed Ras
FDR: false discovery rate
FEV1: forced expiratory volume in 1 s
FOSL1: FOS-like antigen 1
GAB3: GRB2-associated binding protein 3
HV: healthy volunteers
LPS: lipopolysaccharide
Lys - ASA: lysine aspirin
MA: mast cells
MARVELD1: MARVEL domain containing 1
NSAIDs: nonsteroidal anti-inflammatory drugs
PARVG: parvin, gamma
PBMCs: peripheral blood mononuclear cells
PGE2: prostaglandin E2
PLA2: phospholipase A2
qPCR: quantitative PCR
RSV: respiratory syncytial virus
RXRG: retinoid X receptor gamma
SPTBN2: spectrin
TGFB1: transforming growth factor, beta 1
TLR7: toll-like receptor 7
TRIP6: thyroid hormone receptor interactor 6
