# Supplementary material for: The whole - genome expression analysis of peripheral blood mononuclear cells from aspirin sensitive asthmatics versus aspirin tolerant patients and healthy donors after in vitro aspirin challenge
Source: Respir Res. 2015 Dec 9;16:147. doi: 10.1186/s12931-015-0305-4 (PMC4673746; doi:10.1186/s12931-015-0305-4)
Supplement: Additional file 1. — Western Blot protocol. (PDF 176 kb) [file 12931_2015_305_MOESM1_ESM.pdf]

Methods and materials

Western Blot protocol

NuPAGE° Bis-Tris Gels (Life Technologies) were used to separate proteins (200V, from 110 mA – start to 70 mA –end) which were then transferred onto nitrocellulose membrane (1.5h; 30V, from 170 mA – start to 110mA - end)). To block non-specific binding, 5% non-fat dry milk diluted in TBST (0.01% Tween 20 in TBS) was used. After blocking at room temperature for 1 h, the membranes were washed three times with TBST and then gentle agitated with primary antibody dilution buffer containing primary antibodies diluted in TBST with 5% BSA overnight at 4°C. Next, membranes were washed three times in TBST again and blotted with alkaline phosphatase - conjugated anti-rabbit antibodies (Sigma, catalog number: A3687) at a dilution of 1 : 30000 for 1.5 h at room temperature. After incubation with secondary antibodies and triple washing in TBST once again, colorimetric detection of bands by means of BCIP/NBT Alkaline Phosphatase Substrate (Millipore) was performed following the manufacturer's instructions. Densitometric analysis was realized by ImageJ software (v.1.47, NIH, USA).
